# Generation of aroE overexpression mutant of *Bacillus megaterium* for the production of shikimic acid

**DOI:** 10.1186/s12934-015-0251-3

**Published:** 2015-05-17

**Authors:** Saptarshi Ghosh, Uttam Chand Banerjee

**Affiliations:** Department of Pharmaceutical Technology (Biotechnology), National Institute of Pharmaceutical Education and Research, Sector 67, S. A. S., Nagar, 160062 Punjab India

**Keywords:** Shikimic acid, Recombinant *Bacillus megaterium*, Shikimate dehydrogenase, Fermentation

## Abstract

**Background:**

Shikimic acid, the sole chemical building block for the antiviral drug oseltamivir (Tamiflu®), is one of the potent pharmaceutical intermediates with three chiral centers. Here we report a metabolically engineered recombinant *Bacillus megaterium* strain with aroE (shikimate dehydrogenase) overexpression for the production of shikimic acid.

**Results:**

In a 7 L bioreactor, 4.2 g/L shikimic acid was obtained using the recombinant strain over 0.53 g/L with the wild type. The enhancement of total shikimate dehydrogenase activity was 2.13-fold higher than the wild type. Maximum yield of shikimic acid (12.54 g/L) was obtained with fructose as carbon source. It was isolated from the fermentation broth using amberlite IRA-400 resin and 89 % purity of the product was achieved.

**Conclusion:**

This will add up a new organism in the armory for the fermentation based production which is better over plant based extraction and chemical synthesis of shikimic acid.

**Electronic supplementary material:**

The online version of this article (doi:10.1186/s12934-015-0251-3) contains supplementary material, which is available to authorized users.

## Background

Shikimic acid, with three chiral centers is regarded as a versatile hydroaromatic intermediate for the pharmaceutical industry. Since the synthesis of Oseltamivir (Tamiflu^R^) from shikimic acid, its industrial demand has increased exponentially [1]. Due to its unique structure, it has been used as the building block for the synthesis of several biologically active compounds such as antibiotics, antitumor agents [2], antithrombotic agents [3, 4], and vitamins etc. It has also been used in the organic synthesis and cosmetic industry [5]. Currently, shikimate is mainly produced by chemical synthesis or extraction from the fruit of *Illicium* spp. However, these processes are complicated with the high cost and/or limitations of raw materials making it difficult to meet the increasing worldwide requirements due to the global pandemic of influenza [6]. Microbial fermentation is regarded as a potential alternative for large scale production considering the increasing demand of shikimate [7].

**Fig. 1:**
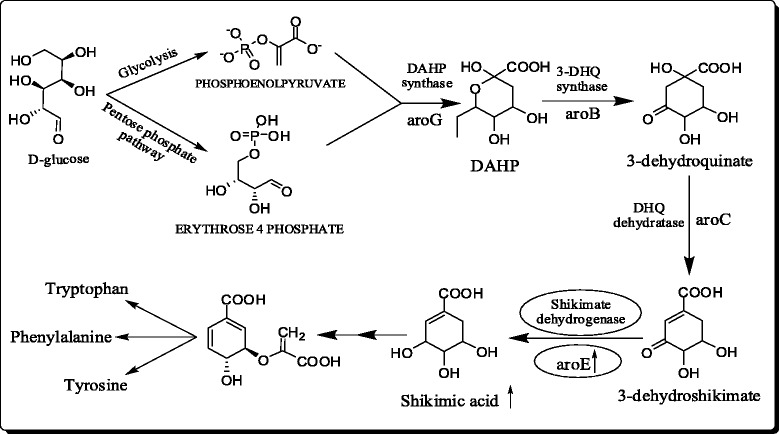
Biosynthetic pathway of shikimic acid [indicating the over expression of aroE gene and enhancement in shikimic acid yield]

Shikimate pathway is responsible for the synthesis of aromatic amino acids and several aromatic compounds in microbes and plants [8, 9]. The shikimate pathway (Fig. 1) starts with the formation of 3-deoxy-D-arabino-heptulosonate-7-phosphate (DAHP), which is formed by DAHP synthase isoenzmyes (aroF, aroG, and aroH). The DAHP so formed, is further catalyzed by three enzymatic steps (aroB, aroC and aroE) to form shikimic acid. Shikimic acid is further transformed into aromatic amino acids (phenylalanine, tyrosine, and tryptophan) through chorismic acid. *Escherichia coli* have been the major target of metabolic engineers and molecular biologists to modify the shikimic acid pathway to have higher concentration of shikimic acid in the fermentation broth. [6, 10–12]. Modification of the central carbon metabolism has been one of the major strategies for the production of shikimic acid by fermentation. Inactivation of the PTS operon (PTS−), expression of non-PTS glucose transporters like glucose facilitators (glf), glucokinase (glk) in combination with over expression of tktA gene was reported to increase the shikimic acid titer to 71 g/L [13]. Antisense RNA interference and gene deletion were employed to inactivate the aroK gene in a shikimic acid producing *E. coli* strain with shikimic acid yield of 1.85 g/L using glycerol as carbon source in a 10-L fermenter [14]. Based on the inactivation of ptsHIcrr, aroK, aroL, pykF and lacI genes in *E. coli*, Rodriguez et al. [12] constructed a high-yield (43 g/L) shikimic acid strain through constitutive expression of selected genes from the pentose phosphate and aromatic pathways. Recently, a modified *E. coli* strain has been reported where aroK knock out mutant was combined with enhanced phosphoenolpyruvate level and a shikimic acid yield of 14.6 g/L was achieved [15]. Chemically inducible chromosomal evolution and cofactor metabolic engineering was reported to have shikimic acid yield of 3.12 g/L [16]. In addition to recombinant *E. coli*, genetically modified *Bacillus subtilis* [17] and *Citrobacter freundii* [18] have been successfully used to overproduce shikimic acid although the titers have not exceeded more than 20 g/L. Statistical modelling approach was used for media optimization and shikimic acid titer of 16.78 g/L was achieved with *C. freundii* [19]. In one of the recent reports, genetically modified *B. subtilis* strain with combined overexpression of genes for 3-deoxy-D-arabinoheptulosonate-7-phosphate synthase (aroA) and SA dehydrogenase (aroD) was mentioned with shikimic acid yield of 3.2 g/L [20]. A pyruvate kinase deficient strain of *B. subtilis* was also reported with shikimic acid yield of 4.67 g/L [21].

In this study, we have developed a modified strain of *Bacillus megaterium* with aroE (shikimate dehydrogenase) overexpression for the production of shikimic acid (Fig. 1). The aroE gene is targeted for this study, as shikimate dehydrogenase is one of the rate limiting steps in the shikimic acid pathway. Various carbon sources were used to study their effect on the yield of shikimic acid. As no computational model was available for shikimate dehydrogenase of *B. subtilis*, a homology model was developed. To rationalize the model, docking study was performed using dehydroshikimate as substrate.

## Results and discussion

### Expression profile of shikimate dehydrogenase and PMF based identification

**Fig. 2:**
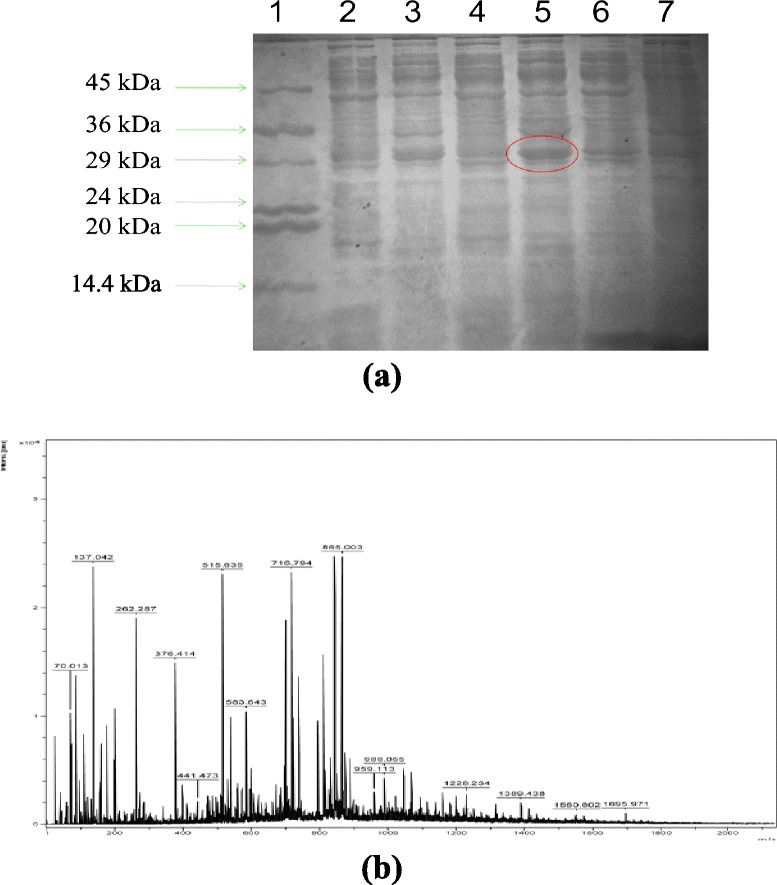
aroE overexpression and peptide mass fingerprinting **(a)** SDS-PAGE (12 %) showing over expression of shikimate dehydrogenase (aroE). Lane 1: Protein marker; Lane 2 & 3: supernatant & pellet of 2 % induction; Lane 4 & 5: supernatant & pellet of 3 % induction; Lane 6 & 7: supernatant and pellet of uninduced sample. **(b)** Peptide profile of the overexpressed protein after in-gel digestion

To generate an aroE overexpression mutant of *B. megaterium*, the aroE gene from *B. subtilis* was cloned into pWH1520 vector. The vector construct was transformed into wild type *B. megaterium* cells for expression. As the vector has a xylose inducible promoter, xylose was fed into the media for induction. Upon optimization, induction with xylose (2 %, w/v), a clear band was observed in the SDS gel (12 %, w/v), above the 29 kDa band of the marker (Fig. 2a). The band was cut from the gel and used for the in-gel digestion. The digested sample was given for MALDI analysis and peptide profile was obtained (Fig. 2b). The peptide mass (*m/z*) values were compared with the MASCOT database and score of > 25 was obtained. These were some of the matched fragments of specific cleavage: 147.138, 175.180, 395.493, 522.910, and 721.747 along with several matches of non-specific cleavages.

### Generation of homology model and docking study

**Fig. 3:**
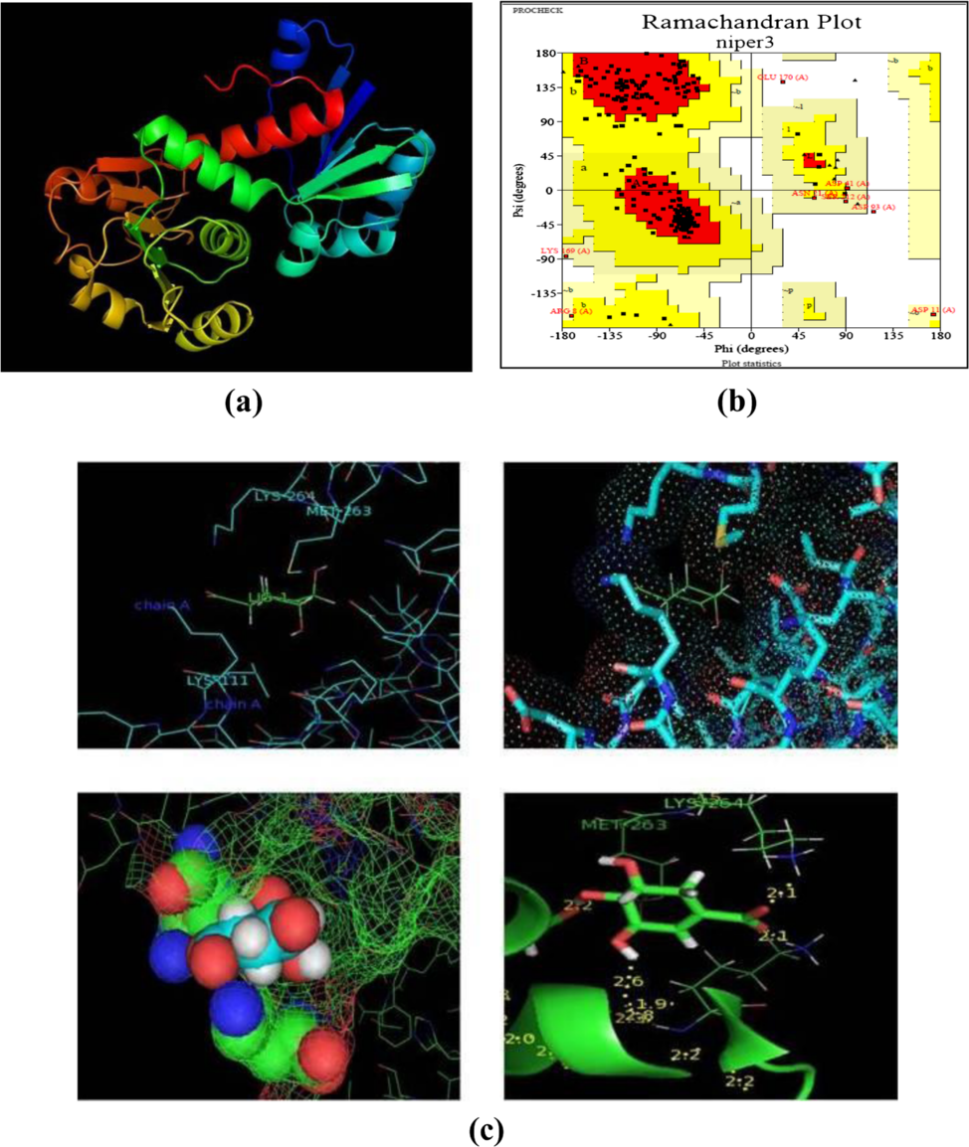
Computational study of shikimate dehydrogenase structure and Docking analysis **(a)** Homology model of shikimate dehydrogenase of *B. subtilis*. **(b)** Ramachandran plot of shikimate dehydrogenase showing the distribution of amino acids. **(c)** Docking studies showing catalytic interaction of shikimate dehydrogenase with dehydroshikimate

To characterize the overexpressed protein, BLAST analysis of the cloned gene sequence was carried out [22]. It showed the presence of NAD(P) binding site in the protein and also confirmed it as a member of the shikimate dehydrogenase superfamily. Sequence alignment demonstrated that it has 98 % similarity with shikimate dehydrogenase of *B. subtilis* subsp. spizizenii str. W23 and 94 % similarity with *B. subtilis* subsp. subtilis str. 168. A homology model was developed for shikimate dehydrogenase of *B. subtilis* MTCC 441 (Fig. 3a) and the corresponding Ramachandran plot was generated (Fig. 3b). From the plot it was found that 83.5 % amino acids are in the most favoured regions (A, B, L) which make it a standard model for shikimate dehydrogenase. Since, there was no such model available for shikimate dehydrogenase of *B. subtilis*; this gives an insight to the structure activity relationship of this enzyme.

Docking studies were carried out with dehydroshikimate as ligand to show the binding interaction of substrate with the enzyme. The pocket finder analysis showed that Lys 111, Met 263, Lys 264 play the crucial role in substrate binding and activity (Fig. 3c).

### Shikimate dehydrogenase activity with aroE overexpression strain

**Fig. 4:**
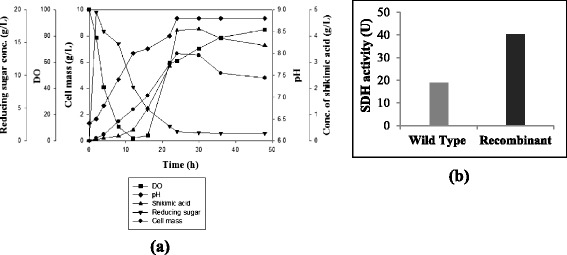
Shikimic acid production using the recombinant strain of *B. megaterium* **(a)** Course of growth and shikimic acid production in a 7 L reactor, **(b)** Shikimate dehydrogenase activity of recombinant strain with respect to the wild type

Shikimate dehydrogenase activity plays a key role in the shikimic acid production by converting dehydroshikimate to shikimic acid. The specific activity of shikimate dehydrogenase was estimated in crude extract of recombinant *B. megaterium*. The wild type strain (*B. megaterium* MTCC 428) was used as a control. From Fig. 4, it is evident that the specific activity of shikimate dehydrogenase in the recombinant strain had increased by 2 fold compared to the wild type. This enhancement in the total activity of shikimate dehydrogenase was due to the aroE overexpression in the recombinant strain.

### Shikimic acid production using recombinant strain

To evaluate the effect of aroE overexpression on the growth and shikimic acid production, a batch reactor of 7 L capacity was run in LB medium with the recombinant *B. megaterium*. The cells were induced with 2 % xylose, when the OD_600nm_ reached to 0.4 and cell mass was 0.8 g/L. During the growth and production of shikimic acid by the recombinant organism, (Fig. 4) reducing sugar concentration started decreasing from the very beginning and it was found to be 1.6 g/L at 24 h. Xylose was used for induction and it seems that the most of the xylose had been utilized by *B. megaterium* for the growth and shikimic acid production. The maximum shikimic acid concentration was 4.2 g/L at the end of 24 h as analyzed and quantified by HPLC. DO concentration showed the downward trend initially along with the increase of growth and finally it reached to the saturation level at the end of fermentation. The initial pH of the fermentation medium was 6.5 and during course of growth and shikimic acid production, it was found that the pH of the fermentation broth was on the increasing side. The cell mass concentration started increasing from the very beginning and a maximum of 6.6 g/L cell mass was obtained at 24 h.

Various biochemical engineering parameters such as volumetric productivity, specific product formation rate and yield are calculated both for the wild type and recombinant strains of *B. megaterium*. It is evident from Table 1 most of the parameters increased many times than the wild type strain. The most important parameter is the volumetric productivity, which is eight times more in the case of recombinant strain than that of the wild type. LB medium as such did not have any carbon source, except the xylose which was used as inducer. It was thought that the addition of extra carbon source in the media might have significant effect on shikimic acid yield. Various carbon sources were fed into the fermentation medium before the inoculation.

**Table 1:** Comparison of fermentation parameters of the wild type and recombinant strain in the growth and production of shikimic acid

Condition	Q_v_ (g SA/L.h)	Q_p_ (mg SA/g cell mass.h)	Y_(X/S)_ (g/100 g)	Y_(P/S)_ (g/100 g)
Wild type	0.01	8.83	25	2.65
Recombinant	0.08	26.31	33.25	21
Fold	8	2.97	1.33	7.92

Q_V_ = Volumetric productivity of shikimic acid

Q_P_ = Specific product formation rate

Y_(X/S)_ = Cell mass yield

Y_(P/S)_ = Product yield w.r.t substrate

### Effect of carbon sources

**Fig. 5:**
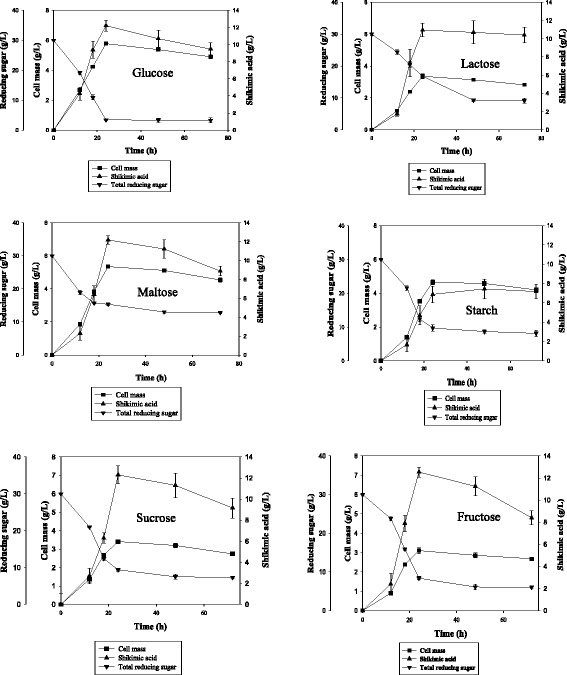
Fermentation profile and shikimic acid yield of recombinant strain under different carbon sources

The effect of various carbon sources (glucose, maltose, sucrose, fructose, lactose, starch) on the growth and shikimic acid production by aroE over-expressing strain of *B. megaterium* was investigated to select the best carbon source (Fig. 5). The cell mass produced in the culture medium under different carbon sources was investigated. The highest amount of cell mass was achieved with glucose as carbon source. Out of the six carbon sources used, the recombinant strain had the highest amount of shikimic acid accumulation when fructose was used as carbon source with the lowest amount of cell mass.

The growth of cell mass and the amount of shikimic acid produced with various carbon sources are shown in Table 2. The shikimic acid yield with various carbon sources was also calculated. Although glucose is a highly metabolizable carbon source, the highest shikimic acid yield (4.04 g/g_DCW_) was observed with fructose. Substrate diversion was more towards the product formation when fructose was used as a carbon source. These data indicate the non-growth linked character of shikimic acid production with fructose as a carbon source, as there are instances of higher growth with other carbon sources than fructose, however, the shikimic acid yield was superior with 1 % fructose.

**Table 2:** Shikimic acid yields with respect to cell mass under different carbon sources

Carbon sources^a^	DCW (g/L)	Shikimic acid (g/L)	Y_(P/X)_
Glucose	5.78	12.21	2.11
Lactose	3.36	10.95	3.25
Maltose	5.35	12.18	2.27
Starch	4.65	6.89	1.48
Sucrose	3.41	12.30	3.6
Fructose	3.1	12.54	4.04

Y_(P/X)_ = Product yield w.r.t cell mass

^a^Carbon sources such as Glucose, Lactose, Maltose, Starch, Sucrose and Fructose (1 %, w/v) were used

### Isolation of shikimic acid and its identification

Anion exchange resin, amberlite IRA-400 Cl^−^ was used for the extraction of shikimic acid from the fermentation broth following the method as described in methods section. After extraction, the product was characterized as shikimic acid by HPLC, Mass and NMR (Additional file 1: Supporting information: S1, S2, S3, S8, S9, S10).

## Conclusion

The effect of overexpression of aroE gene (shikimate dehydrogenase) on the yield of shikimic acid is reported in this paper. The maximum shikimic acid yield of 12.54 g/L was achieved using the recombinant strain at 7 L reactor level. The effect of various carbon sources was investigated and fructose was found to be the most effective carbon source for shikimic acid accumulation. The homology model developed for shikimate dehydrogenase has been used for the docking study and the enzyme-substrate interaction is shown. The amino acid residues involved in substrate binding and catalysis were identified. The study shows that over expression of aroE gene has significant effect over the shikimic acid yield in *B. megaterium* than the previously reported strains. By this single alteration in the pathway, an improved *Bacillus* strain is developed which is better over some of the recombinant counterparts with multiple modifications. Though the yield is lower than the reported values, with further modifications of the recombinant strain (such as generation of aroK knock out), this may be developed as a potential process for industrial application. There is a possibility of having higher titer of shikimic acid on the achievement of complete optimization studies with the recombinant *B. megaterium.*

## Methods

### Culture and growth condition

*B. megaterium* MTCC 428 and *B. subtilis* MTCC 441 was obtained from Microbial Type Culture Centre, Institute of Microbial Technology, Chandigarh, India. *E. coli* Top10, available in the laboratory, was used as cloning host. Luria Bertani broth (10 g/L tryptone, 5 g/L yeast extract, 10 g/L NaCl) was used for the growth of both the organisms. Various concentrations of antibiotics (ampicillin 100 μg/mL, tetracycline 10 μg/mL) was used in the medium for the selection of recombinants of *E. coli* and *B. megaterium,* respectively. Cells were grown in an incubator shaker (Kuhner, Germany) at 37 °C (200 rpm) for 12 h (*E. coli*) and 24 h (*B. megaterium*).

#### ➢ Fermentation media and growth condition

For the production of shikimic acid using wild type and recombinant strain, in shake flask and bioreactor, Luria Bertani broth was used. Induction was carried out with 2 % xylose (w/v) at OD_600nm_ of 0.4 (cellmass 0.8 g/L). Tetracycline (10 μg/mL) was used for the selective growth of the recombinant. For shake flask experiments, the strain (glycerol stock stored at −80 °C) was grown in 20 mL LB-Tet medium as starter culture. This fresh culture was used to inoculate (2 %, v/v) 100 mL medium at 37 °C (200 rpm). For 7 L bioreactor (5 L working volume), 250 mL starter culture was prepared for inoculation. Glucose feeding of 50 g/L was given after 24 h, in the stationary phase. The reactor was aerated at 0.5 VVM and the dissolved oxygen concentration was maintained above 30 % air saturation by agitation at 200–500 rpm. Antifoam (polypropylene glycol) was added manually as and when needed.

### Plasmid construction

**Fig. 6:**
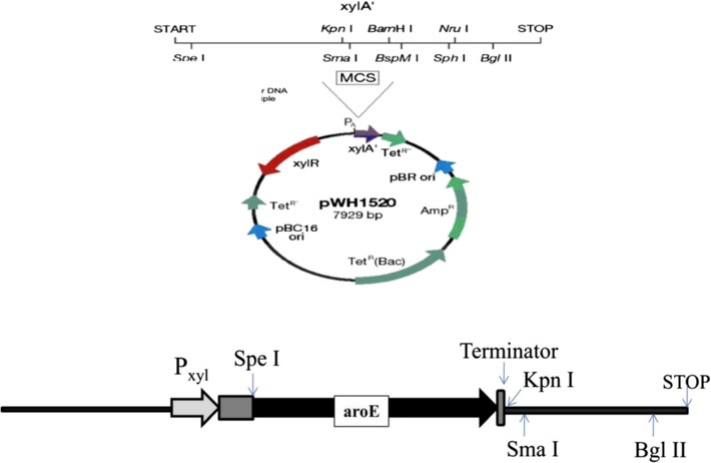
Vector map and plasmid construct

Standard procedures were followed for polymerase chain reactions (PCR), DNA purifications, enzyme digestions, ligation reactions and plasmid extractions [23]. The plasmid construct was verified by PCR using the construct as template, restriction digestion analysis, and sequencing. Wild type gene aroE, from *B. subtilis* MTCC 441, was amplified with primers aroE-FP and aroE-RP, and cloned into plasmid pWH1520 via *Spe*I and *Kpn*I sites to construct plasmid pWH1520-aroE^wt^. Strategy and map of plasmid construct is displayed in Fig. 6.

aroE-FP: 5′ GATGCACTAGTATGAAAAAGCTGTACGGGGTTATCGG 3′

aroE-RP: 5′ GATGCGGTACCTTAACATTCTGTTCCTCCTAATTTTCC 3′

### Transformation of vector construct into protoplast of *B. megaterium*

The transformation of *B. megaterium* protoplasts provides an elegant method to introduce foreign plasmid DNA [24]. For the transformation of protoplasts, method reported by Biedendieck et al. [25] was followed. After transformation, the protoplasts were platted on Tet-LB-agar plate and incubated at 37 °C. Colonies were observed indicating the successful transformation.

### Overexpression of shikimate dehydrogenase (aroE)

In plasmid pWH1520, the promoter is under the control of xylose induction. For the expression of cloned gene, xylose concentration was optimized. Different concentrations of xylose (1, 2, 3, 4, 6 %, w/v) were used. Cells were induced at OD_600nm_ of 0.2-0.4. Cells were harvested after 24 h, once stable OD_600nm_ was achieved. Cells were washed twice with phosphate buffer (100 mM, pH 7.0) and resuspended in lysis buffer (100 mM Tris–HCl, 0.4 mM DTT, 1.2 mM PMSF, 1 mM EDTA) at final cellmass concentration of 100 mg/mL. Cells were disrupted by French Press at 600 psi and the suspension was centrifuged to remove the cell debris. Both the pellet and supernatant was used as sample for PAGE (12 %) to check for the protein.

### Identification of over expressed protein

The over expressed protein was characterized by peptide mass fingerprinting using in-gel digestion method, as reported by Shevchenko et al. [26]. The peptide profile was checked by MALDI analysis and compared with the MASCOT database [27]. The MASCOT score was used for the identification of protein.

### Homology modelling of shikimate dehydrogenase and docking study

Homology model was developed on the basis of sequencing data of the cloned aroE gene using the Expasy server and Swiss Dock model [28]. The model was validated by Prochek. Docking was carried out with Swiss Dock system using dehydroshikimate (from Zinc database) as ligand. Binding site of the ligand was predicted using pocket finder and docking analysis. Binding interaction of the ligand with the enzyme was predicted using Chimera map.

### Assay for shikimate dehydrogenase (SDH)

The enzyme was assayed in the reverse direction using shikimic acid as substrate at 25 °C by monitoring the reduction of NAD^+^ at 340 nm [29]. The assay mixture (total volume 1 mL) contained 100 mM Na_2_CO_3_ (pH 10.6), 4 mM shikimic acid and 2 mM NAD^+^. Substrate blank, enzyme blank and co-factor blank was used for each assay experiment. One unit of enzyme activity is defined as the amount of enzyme that catalyses the conversion of 1 μmol of substrate/min.

### Analytical methods

Cell growth was monitored by measuring the optical density at 600 nm. Glucose concentration was estimated by DNS method [30]. Shikimic acid concentration was determined by HPLC using Waters Alliance e2695 series instrument and Alltech OA-2000 organic acid column (100 × 6.5 mm, 6.5 μm) (Grace Davison Discovery Science, Deerfield, Illinois, USA) maintained at 30 °C. The mobile phase was 0.005 N H_2_SO_4_ with a flow rate of 0.5 mL/min. Shikimic acid was detected at 254 nm with a photodiode detector and quantified using a standard curve.

### Isolation of shikimic acid from fermentation broth

Shikimic acid being anionic in nature, an anion exchange chromatography was used for its extraction from the fermentation broth. Amberlite IRA-400 Cl^−^ was used for this purpose [31]. Cells were separated by centrifugation; supernatant was concentrated on rotavapor till a viscous mass was formed, dissolved in methanol and centrifuged again. The resultant solution was then dried in rotavapor till it formed a layer. The solid residue then re-dissolved in water and filtered. The filtrate was loaded onto an Amberlite IRA-400 chloride column and washed with deionized water. Shikimic acid was eluted with 25 % aqueous acetic acid and concentrated on rotavapor. The concentrate was analyzed by HPLC, GC-MS and NMR.

## Additional file

Additional file 1:
**Supporting information.**
