# Supplementary material for: Generation of aroE overexpression mutant of Bacillus megaterium for the production of shikimic acid
Source: Microb Cell Fact. 2015 May 17;14:69. doi: 10.1186/s12934-015-0251-3 (PMC4490670; doi:10.1186/s12934-015-0251-3)
Supplement: Additional file 1: — Supporting information. [file 12934_2015_251_MOESM1_ESM.docx]

**Supporting information**

**S.1 HPLC chromatogram of standard shikimic acid (flow rate 0.3 mL/min)**

**S.2 HPLC chromatogram of 24 h sample from broth of recombinant *B. megaterium***

**S.3 HPLC chromatogram after extraction**

**S.4 Sequence of aroE gene cloned from *B. subtilis***

AACGCATCATTGAAAGATCTCAGTCTAGACGGACATTACCATGCCTTCAAAGTAGAAGAGGATGATCTGGAAGATGCGGTAAAAGGAATAAGAGCACTCGGCGTACATGGAATCAATGTCACTGTTCCGCATAAGGTTTCCATTATGGATTATCTAGATCATATTGATGATAGTGCAAAAGTGATTGGTGCCGTAAACACGGTCAGAAGAGAGGGAGACAAGCTTGTCGGATACAATACCGACGGGGAAGGCTTTGTGAAGTCATTAATGAAGGTATTGGACAAGCCCATCTCTGAACTG TCATTCTTAATGATCGGCGCGGGCGGAGCGGCAAGAGCCATCTTTACAACAATTGCCCGCAATGTCCCGAAAAAGTTTGACATTTGCAACCGGACGTTGGAAAAAGCAAAGCAGCTTACAGAATCCACTCCCTCGTTTCACAATAAAGAAGTGTTAAGCATAAAAGAAGCAGAAGAACGGCTTAAGCAGTATGATGTAATTATCCATACAACGTCTGTGGGCATGT

ATCCGAACGTGGACGAGGTGCCGATTTCATTACAGCGTGCCGCAAGCAGTGCCGTCGTATGTGATATCGTGTATAATCCAATTCAGACGGCCCTTTTAAAAGAAGCAAACCAAAAAGGCCTGAAAACACTCGATGGAGTGGGCATGTTTGTTGAACAGGCAGCATTGTCTTTCCAGCTATGGACTGGACAAGAGCCTGATATGAAAAAATGAGATCAATGCATTAAAATCGGGAGGAACAGAATGTTAA

**S.5 BLAST analysis of cloned gene**

**
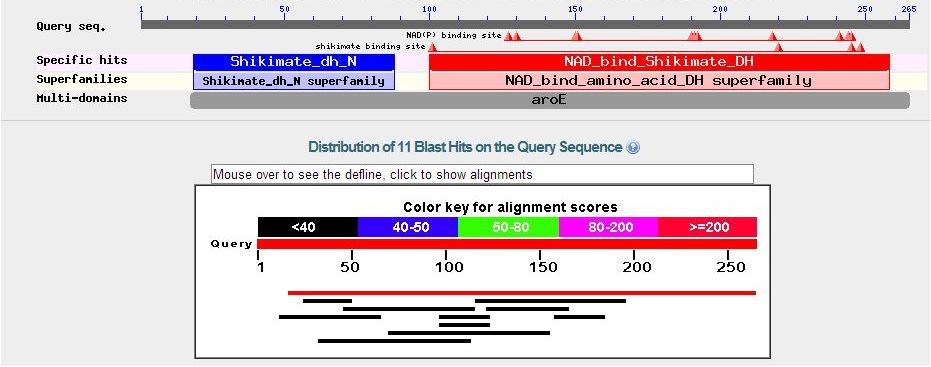
**

**S.6 Standard curve of Shikimic acid**

**
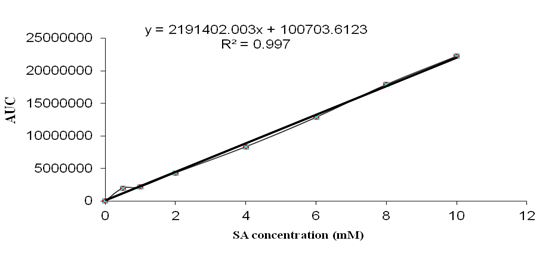
**

**S.7 HPLC chromatogram of carbon source optimization (flow rate: 0.5 mL/min)**

**(a) Sucrose**

|  | Retention Time | Area | % Area | Height |
| --- | --- | --- | --- | --- |
| 1 | 6.299 | 34639186 | 100.00 | 982037 |

**(b) Starch (soluble)**

|  | Retention Time | Area | % Area | Height |
| --- | --- | --- | --- | --- |
| 1 | 6.276 | 30033767 | 100.00 | 880289 |

**(c) Maltose**

|  | Retention Time | Area | % Area | Height |
| --- | --- | --- | --- | --- |
| 1 | 6.225 | 34226804 | 100.00 | 850392 |

**(d) Lactose**

|  | Retention Time | Area | % Area | Height |
| --- | --- | --- | --- | --- |
| 1 | 6.249 | 30605235 | 100.00 | 781991 |

**(e) Glucose**

|  | Retention Time | Area | % Area | Height |
| --- | --- | --- | --- | --- |
| 1 | 6.257 | 26346085 | 100.00 | 839867 |

**(f) Fructose**

|  | Retention Time | Area | % Area | Height |
| --- | --- | --- | --- | --- |
| 1 | 6.266 | 35247087 | 100.00 | 583982 |

**S.8 Mass spectra of sample after extraction showing the peak of shikimic acid**

**
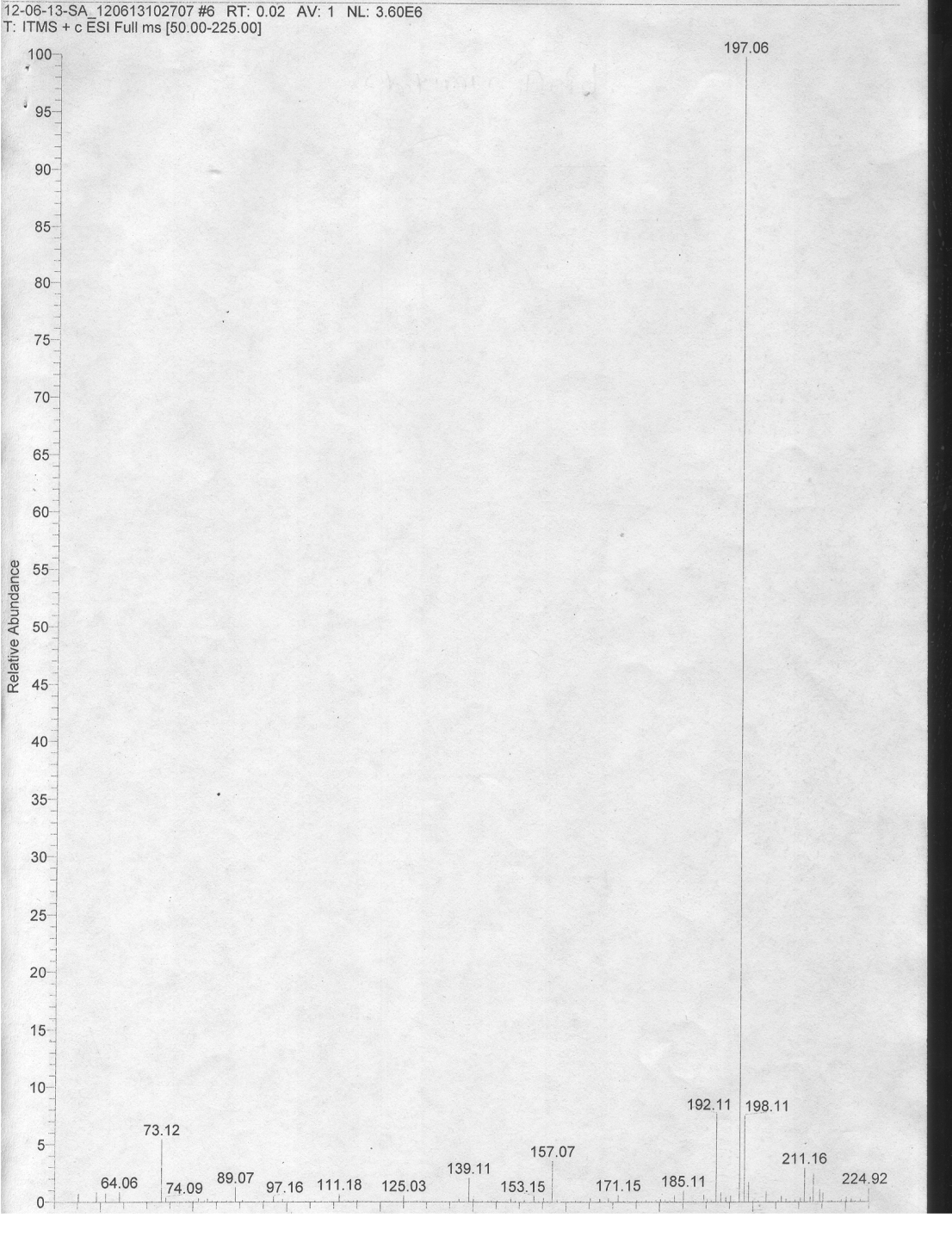
**

**S.9 ^1^H NMR spectra of isolated product (shikimic acid) from the reaction mixture**

**
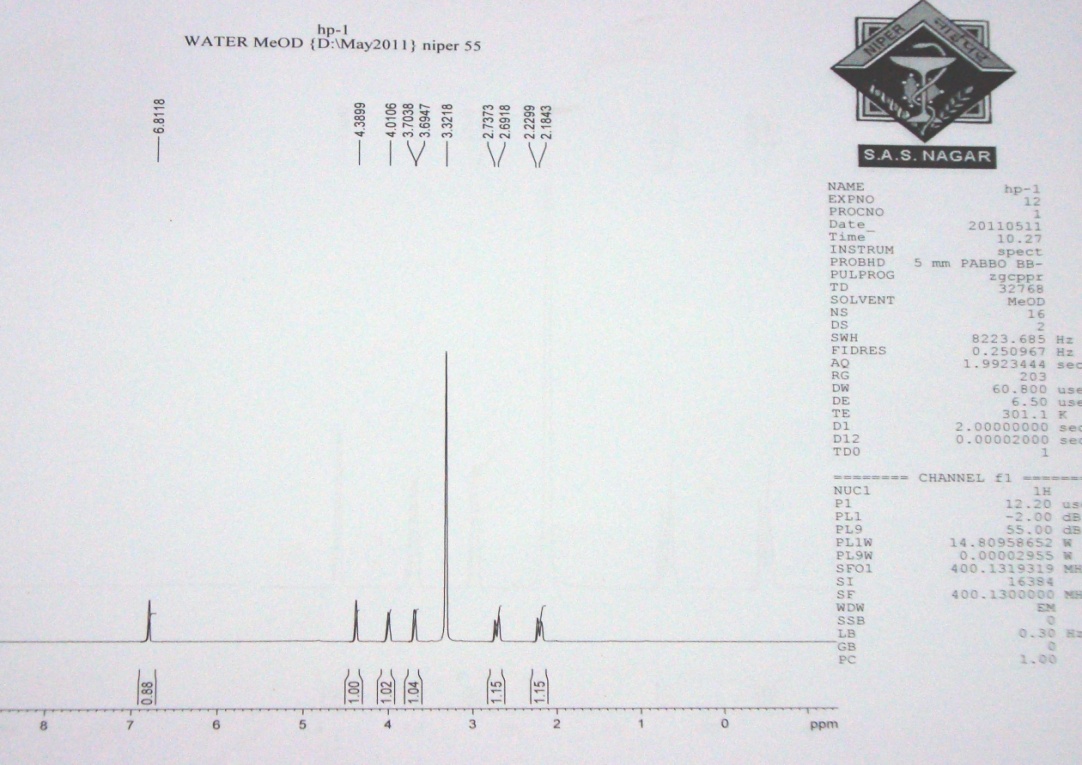
**

**S.10 ^13^C NMR spetra of isolated product (shikimic acid) from the reaction mixture**

**
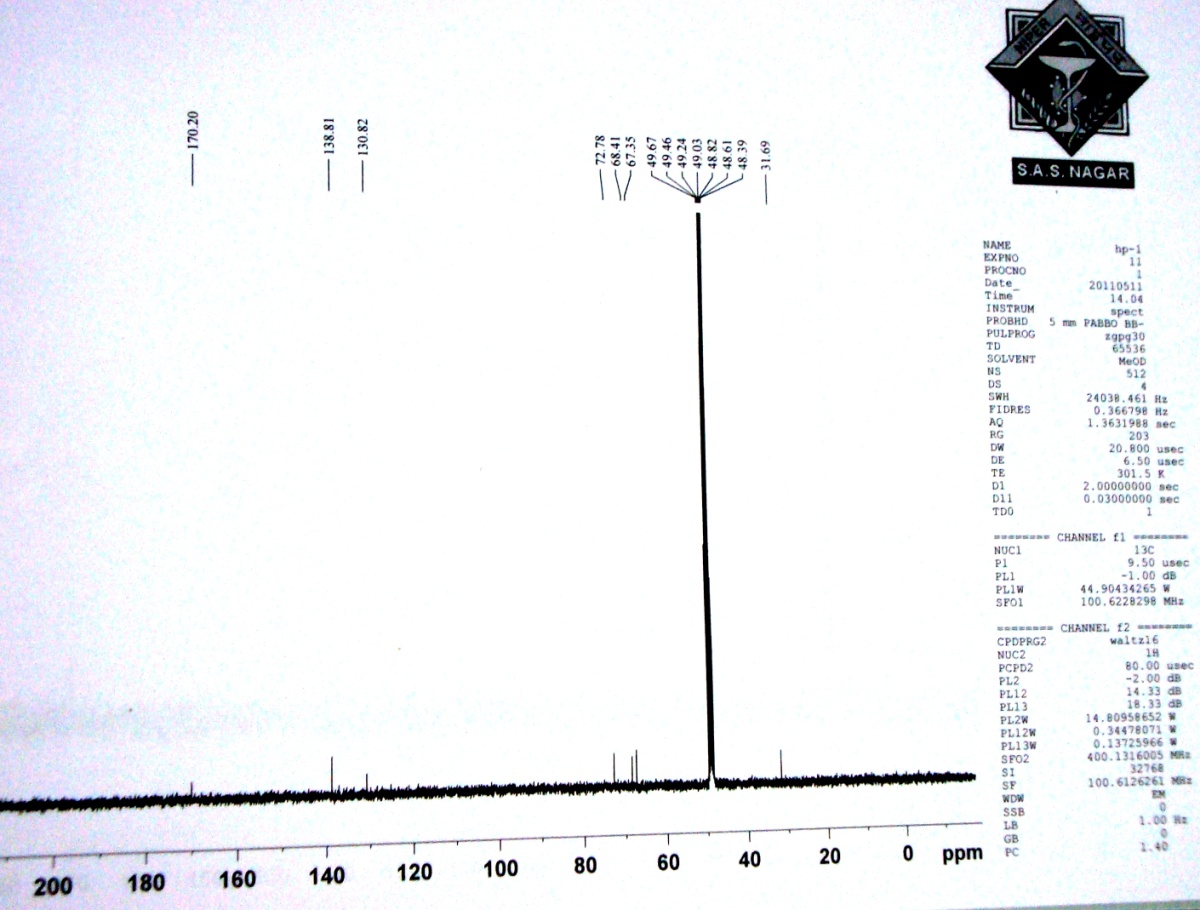
**
